# Spatial and temporal intratumoral heterogeneity in breast cancer: a systematic and conceptual review of single-cell and spatial omics studies

**DOI:** 10.1186/s12885-026-15928-0

**Published:** 2026-04-06

**Authors:** Imad Barjij, Oumaima Lamsyah, Sanae Kdadri, Sihame Lkhoyaali, Salma Najem, Sarah Naciri, Hanane Inrhaouen, Ibrahim Elghissassi, Saber Boutayeb, Hind Mrabti, Hassan Errihani

**Affiliations:** Medical Oncology Department, National Institute of Oncology, Ibn Sina University Hospital, Faculty of Medicine and Pharmacy of Rabat, Mohammed V University, Rabat, Morocco

**Keywords:** Breast cancer, Intratumoral heterogeneity, Single-cell RNA sequencing (scRNA-seq), Spatial transcriptomics, Tumor microenvironment, Clonal evolution, Subclonal plasticity, Temporal dynamics, Multi-omics integration, Therapeutic resistance

## Abstract

**Background:**

Spatial and temporal intratumoral heterogeneity (ITH) remains a major challenge in the diagnosis, prognosis, and treatment of breast cancer. Recent advances in single-cell and spatial omics technologies have enabled unprecedented resolution of subclonal architectures, evolutionary trajectories, and microenvironmental interactions. This systematic and conceptual review aimed to synthesize and integrate current evidence on spatiotemporal ITH in human breast cancer, bridging empirical data with mechanistic interpretation through high-resolution profiling platforms.

**Methods:**

We conducted a systematic review following PRISMA 2020 guidelines, searching three databases (PubMed, Scopus, and Web of Science) and screening 1037 records published between January 2018 and May 2025. 19 original studies were included based on predefined eligibility criteria targeting single-cell RNA sequencing (scRNA-seq), spatial transcriptomics, or multi-omics approaches applied to human breast tumor samples. Data extraction focused on study design, technologies used, subclonal dynamics, spatial/temporal resolution, tumor–immune interactions, and risk of bias.

**Results:**

The included studies analyzed over 400,000 single cells from diverse breast cancer subtypes, with a predominance of triple-negative breast cancer. Subclonal plasticity was a recurrent feature, often characterized by EMT (epithelial-to-mesenchymal transition) signatures, cell-cycle heterogeneity, and immune evasion. Spatial analyses revealed discrete ecological niches shaped by immune exclusion and stromal patterning, while temporal assessments uncovered therapy-driven clonal selection, metabolic reprogramming, and enhancer remodeling. Interclonal and tumor–immune communication were consistently associated with poor prognosis or therapeutic resistance. Most studies were judged to have low or moderate risk of bias, with transparent reporting and accessible data pipelines.

**Conclusions:**

Single-cell and spatial omics studies provide critical insights into the evolutionary ecology of breast cancer. By conceptually integrating spatial, temporal, and microenvironmental dimensions, this review highlights convergent evolutionary programs underlying tumor aggressiveness and resistance. Spatiotemporal ITH is a key driver of disease progression, and its systematic characterization could inform biomarker development, personalized therapies, and future multi-modal diagnostics. Continued integration of spatial, temporal, and functional data is essential to move from descriptive maps to clinically actionable frameworks.

**Supplementary Information:**

The online version contains supplementary material available at 10.1186/s12885-026-15928-0.

## Introduction

Breast cancer is a highly heterogeneous disease, both genetically and phenotypically, and represents a leading cause of cancer-related mortality in women worldwide. Among its subtypes, triple-negative breast cancer (TNBC) poses a particular therapeutic challenge due to the absence of hormone receptors and HER2 amplification, which limits the use of targeted therapies. Over the past decade, next-generation sequencing has revealed a complex genomic landscape within breast tumors. However, traditional bulk RNA-sequencing fails to resolve the cellular and spatial complexity underlying tumor evolution, resistance, and immune evasion [1–4].

Intratumoral heterogeneity (ITH) encompasses the coexistence of genetically, epigenetically, and phenotypically distinct tumor cell populations within the same lesion. It can be structured across both spatial and temporal dimensions, leading to functional consequences such as therapeutic resistance, clonal selection, and immune escape. The tumor microenvironment (TME), composed of immune cells, fibroblasts, vasculature, and microbial components, further adds a layer of complexity by influencing tumor behavior and response to treatment. Understanding the dynamics of ITH and TME interactions is thus critical for improving diagnosis, prognosis, and therapeutic strategies in breast cancer [5–7].

The emergence of single-cell RNA sequencing (scRNA-seq), spatial transcriptomics, and integrative multi-omics technologies has revolutionized our ability to deconstruct tumor ecosystems at unprecedented resolution. These methods allow the profiling of thousands of individual cells, capturing transcriptional states, clonal trajectories, regulatory networks, and spatial architectures within tumors. Spatial omics further maps the localization and interaction of cellular subpopulations, thereby uncovering niche-specific behaviors that would otherwise be lost in bulk analyses [8–11].

Despite the rapid expansion of single-cell studies in oncology, there is still no comprehensive synthesis focusing on how these technologies have contributed to our understanding of spatial and temporal ITH in breast cancer. Existing systematic reviews on breast cancer omics often generalize findings across tumor types or focus on technical benchmarks rather than biological insights. Moreover, few syntheses have attempted to integrate data across spatial transcriptomics, scRNA-seq, and multi-modal single-cell studies, particularly in relation to clonal dynamics and tumor–microenvironment interactions. Given the complexity of cellular hierarchies and the diversity of experimental designs, a rigorous systematic review is essential to consolidate current knowledge, identify gaps, and highlight translational implications.

Several key uncertainties remain in the field. First, while numerous studies have identified subclonal tumor populations, their temporal evolution during treatment or progression is often insufficiently characterized. Second, the integration of spatial and functional data remains limited, restricting our understanding of how microenvironmental cues modulate tumor heterogeneity. Third, there is a need to assess how methodological choices (e.g., cell dissociation, clustering algorithms, or spatial resolution) influence biological interpretations across studies. Therefore, synthesizing high-quality, peer-reviewed studies using validated single-cell or spatial techniques applied to human breast tumors could provide an invaluable resource to the field.

This systematic review directly addresses the need for integrative approaches to unravel tumor complexity through high-resolution profiling. Our objective is to bridge molecular insights with clinical applicability by focusing on functional intratumoral heterogeneity, subclonal architecture, and spatially-defined microenvironmental interactions in breast cancer. The review encompasses both treatment-naïve and post-treatment samples, integrates longitudinal observations where available, and includes functional validations through pseudotime analyses, regulatory network inference, and spatial ligand–receptor mapping.

We also aim to compare and contrast the computational and experimental methodologies employed across studies, providing insight into the reproducibility and generalizability of findings. By critically appraising each study’s design, bias risk, and data depth, we seek to establish a robust synthesis of how spatial and temporal heterogeneity has been investigated over the past seven years in breast cancer research.

The objective of this systematic review is to synthesize and critically analyze evidence from single-cell and spatial omics studies that investigate spatial and/or temporal intratumoral heterogeneity in human breast cancer between 2018 and 2025. We aim to identify the major patterns of tumor heterogeneity and the underlying subclonal architectures uncovered through scRNA-seq, spatial transcriptomics, or integrative multi-omics approaches. We also seek to characterize the dynamic trajectories of both tumor and microenvironmental cells over time, including during therapeutic responses or disease progression, to better understand how cell states evolve. Particular attention is given to the extent and nature of spatial heterogeneity, such as the localization of subclonal populations, immune cell niches, and ligand–receptor signaling circuits within tumor tissues. Furthermore, this review examines the methodological consistency and analytical strategies used across studies, including clustering algorithms, trajectory inference tools, and validation frameworks, to assess their impact on biological interpretations. Finally, we explore the clinical implications of the reported heterogeneity, particularly regarding diagnostic, prognostic, and predictive biomarker development in the context of personalized oncology.

This review is not focused on assessing the effects of a therapeutic intervention per se, and therefore does not follow a PICO framework. Instead, it follows a question-driven approach aligned with emerging concepts in cancer evolution and personalized oncology.

Given this complexity, we adopted a hybrid systematic–conceptual approach to integrate current evidence across spatial and temporal dimensions of breast cancer heterogeneity.

## Methods

### Eligibility criteria

We included peer-reviewed original research articles published between January 1, 2018, and May 30, 2025, written in English, that employed single-cell technologies (scRNA-seq, spatial transcriptomics, or multi-omics at single-cell resolution) to investigate spatial and/or temporal ITH in human breast cancer. Eligible studies had to utilize human tumor samples (fresh biopsies or clinically derived datasets) and explicitly address at least one of the following: subclonal architecture, clonal dynamics, spatial heterogeneity, tumor microenvironmental interactions, or cellular trajectories. We included both treatment-naïve and post-treatment tumor samples. Only studies providing full-text access were considered.

We excluded reviews (systematic or narrative), editorials, letters, protocols without results, as well as studies based solely on animal models or cell lines without human validation. Studies using only bulk transcriptomic or low-resolution genomic data, without a single-cell or spatial component, were also excluded. Articles in languages other than English or lacking accessible full-text were not eligible. Studies not addressing tumor heterogeneity, clonal dynamics, spatial organization, or tumor–microenvironment interactions were considered out of scope.

The 2018–2025 time window was selected to capture the era during which single-cell and spatial omics technologies reached maturity in breast cancer research. Earlier studies, while foundational, relied on bulk sequencing without spatial or single-cell resolution and thus fell outside the scope of this review. A sensitivity check confirmed that including these pre-2018 datasets would not have altered the conclusions, as they lacked the spatiotemporal resolution required to assess intratumoral heterogeneity.

### Information sources

The following databases were searched: PubMed (via NCBI), Scopus (via Elsevier), and Web of Science (via Clarivate). The final search was conducted on May 30, 2025. In addition, we examined the reference lists of all included articles and of relevant reviews for potentially eligible studies. Citation tracking (backward and forward) was performed using Google Scholar. No study registries or unpublished literature were considered.

### Search strategy

A comprehensive search strategy was developed using combinations of controlled vocabulary and free-text terms related to breast cancer, single-cell RNA sequencing, spatial omics, and tumor heterogeneity. Search terms included: “breast cancer,” “single-cell RNA-seq,” “spatial transcriptomics,” “multi-omics,” “tumor heterogeneity,” “clonal evolution,” and “microenvironment.” Filters were applied to restrict results to human studies, English language, and publication years between 2018 and 2025.

The complete Boolean search strings were adapted to each database as follows:

- PubMed (title/abstract): (“breast neoplasms”[MeSH Terms] OR “breast cancer”[Title/Abstract]) AND (“single-cell RNA sequencing” OR “scRNA-seq” OR “spatial transcriptomics” OR “spatial omics” OR “multi-omics”) AND (“heterogeneity” OR “clonal evolution” OR “tumor microenvironment” OR “spatiotemporal”).
- Scopus (TITLE-ABS-KEY): (“breast cancer”) AND (“single-cell RNA-seq” OR “spatial transcriptomics” OR “multi-omics”) AND (“heterogeneity” OR “clonal evolution” OR “microenvironment” OR “temporal”).
- Web of Science (Topic): (“breast cancer”) AND (“single-cell RNA sequencing” OR “spatial transcriptomics”) AND (“heterogeneity” OR “evolution” OR “tumor microenvironment”).

Filters were applied to restrict results to *English language*, *human studies*, and *publication dates between January 1 2018 and May 30 2025*.

The searches were last rerun on May 30 2025 to ensure completeness prior to submission.

Preprints were excluded unless subsequently peer-reviewed and published. Duplicate records across databases were removed using Zotero’s de-duplication algorithm prior to screening in Rayyan.

### Selection process

All records were imported into Zotero for de-duplication and then screened using Rayyan. Two independent reviewers (I.B. and O.L.) screened titles and abstracts for relevance. Disagreements were resolved by discussion or third-party arbitration (S.L.). Full-text articles were subsequently assessed by the same reviewers using the predefined eligibility criteria. The PRISMA 2020 flow diagram summarizes the selection process. No automation tools or machine learning algorithms were used.

This review was conducted according to a pre-specified methodological framework based on the PRISMA 2020 guidelines.

Although the protocol was not formally registered in PROSPERO due to the hybrid conceptual nature of the review (which extends beyond conventional clinical outcomes), a predefined methodological framework was established a priori and strictly adhered to throughout the process. This framework included eligibility criteria, search strings, extraction variables, and bias assessment tools, all described in detail within the Methods section and summarized in Tables 1 and 2. This approach ensured methodological transparency, reproducibility, and traceability equivalent to a formally registered protocol.

**Table 1:** Summary of included studies: models, technologies, objectives, heterogeneity types explored, and main findings

Author / Year	Model Used	Technology Applied	Study Objective	Spatial Heterogeneity	Temporal Heterogeneity	Microenvironment Analysis	Main Findings	Evidence Level	Risk of Bias
Sammut et al. 2022 [12]	Fresh frozen biopsies (human, *n* = 168)	Multi-omics (WES, RNA-seq, digital pathology)	To predict response to neoadjuvant chemotherapy using multi-omic features of the tumor ecosystem	Yes	Partial	Yes	High immune activity and proliferation associated with pCR. Machine learning integrating immune and genomic features predicted response (AUC 0.87).	High (large cohort, multi-layered data, external validation)	Low
Cheng et al. 2024 [13]	Human tumor tissue (scRNA-seq datasets)	scRNA-seq + pseudotime + CNV	To dissect TNBC heterogeneity and identify malignant subclusters and drug targets	Yes	Yes	Yes	Identified malignant epithelial subcluster (E8) with high proliferation; interactions with M2 macrophages and EMT-fibroblasts; pelitinib proposed as therapy	High (multi-cohort, spatial trajectory, functional validation)	Moderate
Davies et al. 2020 [14]	S1 (non-malignant) and T4-2 (malignant) human cell lines	Live-cell imaging + scRNA-seq + ERK biosensors	To explore how paracrine EGFR-RAS-ERK signaling drives dynamic gene expression heterogeneity	Yes	Yes	Yes	Demonstrated that amphiregulin-driven paracrine signaling induces pulsatile ERK activation and transcriptional heterogeneity in basal-like breast cancer cells	High (elegant live-cell + scRNA-seq, mechanistic link proven)	Low
Gao et al. 2024 [15]	406,501 cells from 181 human tumor samples	scRNA-seq + spatial transcriptomics + multi-omics	To map breast cancer subtypes based on single-cell origin patterns and identify new therapeutic targets	Yes	Yes	Yes	Identified LP subtype with high genomic instability, poor prognosis, but sensitivity to NAC, PARPi, and ICBs; PLK1 as subtype-specific therapeutic target	High (large-scale atlas, validated in vivo/in vitro)	Low
Jiang et al. 2021 [16]	Human tumor cells from public datasets (GSE75688, GSE118389)	scRNA-seq + CIBERSORT	To characterize intratumoral heterogeneity and immune infiltration patterns in TNBC	Yes	No	Yes	Identified 5 cell types including tumor and immune subsets; M2 macrophages and neutrophils linked to poor prognosis; intercellular communication explored	Moderate (clear scRNA-seq use, no spatial or temporal dynamics)	Moderate
Karaayvaz et al. 2018 [17]	Fresh tumor biopsies from 6 TNBC patients (868 cells)	scRNA-seq + CNV + expression signature matching	To identify subclonal epithelial states and their relation to aggressiveness in TNBC	Yes	Partial	Yes	Revealed co-existence of cycling and quiescent clones; subclones enriched for stemness or EMT; distinct transcriptional and CNV profiles	High (well-conducted study, functional interpretation of subclones)	Low
Kim et al. 2023 [18]	2 fresh ER+ tumor biopsies (human, treatment-naïve)	scRNA-seq + scATAC-seq	To define male-specific transcriptional and enhancer landscapes in MBC	Yes	Partial	Yes	Identified MBC-specific enhancers for ANXA2 and PRDX4; MYC/mTORC1 pathways enriched; rewiring of 5141 cancer-specific enhancer–gene links	High (matched scRNA/ATAC, enhancer-level insights)	Low
Gao et al. 2024 [19]	HCC1143 TNBC cells (scRNA-seq at 24 h and 72 h paclitaxel)	scRNA-seq + pseudotime + SCENIC	To explore paclitaxel resistance mechanisms using single-cell transcriptomic profiling	Yes	Yes	Yes	Identified 3 resistant subclusters (AKR1C3+, IDO1+, HEY1+); inflammatory and interferon signaling increased with treatment; STAT1, IRF7, CEBPB as key TFs	High (rich analysis, pseudotime + GRNs + validated targets)	Low
Tzeng et al. 2024 [20]	Human tumor biopsies (*n* = 25) + public datasets	scRNA-seq + spatial transcriptomics + multi-omics	To explore the prognostic and immune role of MiCU1/2 in breast cancer	Yes	No	Yes	High MiCU1/2 expression associated with immune infiltration (macrophages), recurrence risk, and specific ligand-receptor signaling networks	High (multi-layered omics + spatial + in situ validation)	Low
Yuan et al. 2023 [21]	MDA-MB-231 cell lines (SP1–SP4), 3132 patients (TCGA, GEO, METABRIC)	scRNA-seq + proteomics + spheroid assay + in vitro/in silico	To identify immune-related stemness genes and explore their interaction with the TIME in BC	Partial	Yes	Yes	Identified LDLR as a key immune-stemness gene; correlates with poor prognosis, immune exclusion, and enhanced stem-like properties via scRNA-seq and proteomics	High (multi-modal, single-cell validated)	Moderate
Zhao et al. 2023 [22]	TCGA (*n* = 960) + validation (GSE176078) + scRNA-seq	Multi-omics (CNV, mutation, methylation, expression) + single-cell	To identify breast cancer subtypes via individual-level multi-omics integration (DDE + REO)	Yes	Yes	Yes	Identified 4 subtypes (Mes, Lum, Gen, Im) with distinct EMT, immune, and genomic instability profiles; validated SDHD (11q23 deletion) as mesenchymal driver	High (novel individual DDE integration + scRNA & functional)	Moderate
Zhao et al. 2025 [23]	15 human tumor samples (*n* = 60 sections)	16 S rRNA, RNAscope FISH/CISH, GeoMx DSP, proteomics	To map the spatial distribution of Fusobacterium nucleatum and its effects on breast tumor biology	Yes	No	Yes	F. nucleatum shows focal colonization in tumor regions; upregulates MAPK signaling, VEGFD, PAK1; promotes proliferation/migration; potential therapeutic targets identified	High (spatial + functional + transcriptomic proteomic profiling)	Low
Regner et al. 2024 [24]	12 primary tumors + 4 normals + cell lines	scRNA-seq + scATAC-seq + inferCNV + LMM modeling	To define subtype-specific cis-regulatory enhancer logic in breast cancer using multi-omics single-cell data	Partial	Yes	Partial	Identified subtype-specific enhancers switching from silencers (normal) to enhancers (tumor); linked HEY1 activation in basal-like BC to novel enhancers	High (deep enhancer-to-gene modeling at single-cell level)	Low
Song et al. 2024 [25]	9 tumor samples (scRNA-seq) + 1111 plasma samples	scRNA-seq (tumor) + untargeted & targeted metabolomics + SVM	To identify early diagnostic and predictive metabolic biomarkers using AI-guided multi-omics integration	Partial	No	Yes	Identified purine/pyrimidine metabolism upregulation in TNBC; inosine and uridine levels predict NAC response; Treg activation linked to A2AR expression	High (robust scRNA + metabolomics + TNBC-focused prediction)	Low
Zhang et al. 2024 [26]	5 primary BC samples (GEO GSE180286)	scRNA-seq + SCENIC + CellChat + functional assays	To identify TNBC-specific epithelial cell subsets and regulatory networks promoting progression	Yes	Yes	Yes	Identified 4 TNBC-specific epithelial subtypes (SCD1+, MKI67+, ANXA3+, AQP5+); ANXA3 + cells suppress T-cell function via multiple pathways; XBP1 and FOS validated as key regulators	High (scRNA-seq + GRNs + CellChat + functional validation)	Low
Zhou et al. 2021 [27]	6 TNBC patients (868 epithelial cells + 240 normal)	scRNA-seq + CNV inference + GRN + CellPhoneDB	To dissect subtype-specific GRNs and identify key transcriptional regulators driving TNBC heterogeneity	Yes	Yes	Yes	Identified 5 molecular subtypes (PAM50) in malignant cells; subtype-specific GRNs showed ETV6 as a central regulator with divergent functions; strong macrophage-TNBC interactions	High (fine-grained GRN + centrality metrics + functional annotation)	Low
Janesick et al. 2023 [28]	Fresh-frozen human breast cancer tissues (luminal, HER2+, and TNBC subtypes, *n* = 35)	Spatial transcriptomics (10x Visium) + scRNA-seq integration + multiplex RNA-FISH	To define spatial transcriptional gradients and immune–stromal interactions shaping intratumoral heterogeneity in breast cancer	Yes (high-resolution mapping of epithelial and immune compartments)	Partial (pseudotemporal analysis of epithelial–mesenchymal transitions and metabolic shifts)	Yes (fibroblasts, macrophages, endothelial, T cells)	Identified conserved spatial programs across subtypes linking epithelial plasticity to localized immunosuppression. Showed spatial coupling of EMT-like epithelial clusters with CAF-rich zones and M2 macrophages. Integration of ST and scRNA-seq uncovered ligand–receptor signaling promoting immune exclusion.	High (multi-platform validation, robust computational integration, subtype diversity)	Low
Wang et al. 2024 [29]	Human breast cancer surgical specimens (invasive ductal carcinoma, *n* = 47)	Spatial transcriptomics (Visium 10x Genomics) + multiplex immunofluorescence (mIF) + scRNA-seq integration	To map spatially resolved gene-expression programs and immune–stromal interactions underlying intratumoral heterogeneity in breast cancer	Yes	Partial (pseudotime and treated vs. naïve comparison)	Yes (immune and stromal compartments)	Identified spatially restricted epithelial subpopulations and immune cell niches driving therapy resistance and metabolic reprogramming. ST integration with scRNA-seq revealed ligand–receptor circuits between tumor cells and fibroblasts mediating immune exclusion.	High (large human cohort, multi-omic integration, cross-validation)	Low
Wu et al. 2021 [30]	Human breast cancer samples (*n* ≈ 100 000 cells, multiple subtypes)	scRNA-seq + Spatial transcriptomics (Visium) + IHC + computational integration	To generate a single-cell and spatially resolved atlas of human breast cancers across molecular subtypes	Yes	Partial (cross-sectional, with inferred differentiation states)	Yes	Identified conserved epithelial, stromal, and immune programs across TNBC, ER+, and HER2 + tumors. Defined spatially organized immune–stromal “ecotypes” predictive of prognosis. Demonstrated subtype-specific localization of tumor–immune niches and transcriptional states.	High (large dataset, multi-technology integration, multi-subtype coverage)	Low

**Table 2:** Risk of bias assessment across included studies, tools used, and domain-specific justifications

Author / Year	Type of Study	Risk of Bias	Assessment Tool	Bias Details
Sammut et al. 2022 [12]	Prospective cohort study using fresh-frozen human biopsies (*n* = 168), multi-omics (WES, RNA-seq, digital pathology), with external validation cohort	Low	ROBINS-I	Bias due to confounding: low (prospective design, uniform treatment setting). Selection bias: low (ultrasound-guided biopsy collection, well-defined inclusion criteria). Bias in classification of interventions: not applicable (observational, no intervention misclassification). Bias due to deviations from intended interventions: low (standardized neoadjuvant chemotherapy protocols). Bias due to missing data: low (complete multi-omics datasets reported). Bias in measurement of outcomes: low (pCR as objective endpoint, centralized pathology). Reporting bias: low (predefined analyses, results validated externally). Overall risk considered low due to methodological robustness and external validation.
Cheng et al. 2024 [13]	Retrospective in silico analysis using public scRNA-seq datasets + in vivo mouse validation (4T1 model)	Moderate	Adapted OHAT + elements from ROBINS-I	Selection bias: moderate (non-random selection from public datasets, possibly non-representative TNBC samples). Performance bias: moderate (lack of randomization or blinding in data acquisition). Detection bias: low (scRNA-seq analysis and clustering performed using standard validated pipelines). Attrition bias: not applicable (no loss to follow-up in silico). Reporting bias: low (comprehensive data shown, matched figures/tables). External validity: moderate (functional validation only in murine model, no human clinical correlation). Overall judged moderate due to selection and indirectness of validation.
Davies et al. 2020 [14]	In vitro mechanistic study using S1 and T4-2 human breast epithelial cell lines, live-cell ERK biosensors, scRNA-seq, and time-lapse imaging to explore dynamic paracrine signaling	Low	SYRCLE’s Risk of Bias tool (adapted for in vitro)	Selection bias: low (well-characterized human-derived cell lines, consistent culture conditions). Performance bias: low (automated and real-time imaging minimizes operator-dependent variability). Detection bias: low (quantitative readouts from biosensors and sequencing). Attrition bias: not applicable (no longitudinal dropout). Reporting bias: low (data presented in full; methods and controls well described). External validity: limited (in vitro only), but not a concern given mechanistic scope. Overall risk is low due to controlled conditions, replicates, and transparent reporting.
Gao et al. 2024 [15]	Large-scale multi-omics study integrating scRNA-seq (406,501 cells), spatial transcriptomics, proteomics, and functional validation (in vitro and in vivo) across 181 breast tumor samples	Low	ROBINS-I + STROBE-omics alignment	Confounding bias: low (diverse sample origins accounted for, robust statistical correction). Selection bias: low (broad sampling across subtypes and stages, *n* = 181 tumors). Classification bias: low (clear transcriptomic clustering, integration with CNV/spatial data). Measurement bias: low (uniform pipelines across omics layers, cross-validation). Missing data: low (minimal dropout reported, large n). Reporting bias: low (extensive supplementary data, clear hypotheses). External validity: high (findings validated in preclinical models). The comprehensive, layered approach with functional backing supports a low risk of bias.
Jiang et al. 2021 [16]	Original study using public scRNA-seq datasets and immune deconvolution (CIBERSORT) to characterize TNBC heterogeneity	Moderate	OHAT (adapted for in silico transcriptomic and immune inference studies)	Selection bias: Moderate, retrospective data from public databases (GSE75688 and GSE118389). Performance bias: Low, standardized pipelines used for scRNA-seq integration (Seurat). Detection bias: Moderate, inference methods (CIBERSORT) may introduce error due to reliance on bulk reference profiles. Confounding: Moderate, lack of control for patient-level variability and subtype-specific contexts. Reporting bias: Low, full methodology and data are transparently reported.
Karaayvaz et al. 2018 [17]	Single-cell transcriptomic profiling of fresh tumor biopsies (*n* = 6 TNBC patients, 868 cells) to investigate subclonal architecture and aggressiveness	Low	OHAT (adapted to single-cell tumor profiling with clinical biopsy origin)	Selection bias: Low, direct fresh tumor sampling from TNBC patients with immediate dissociation and quality control. Performance bias: Low, robust filtering, clustering, and CNV inference applied to remove technical noise. Detection bias: Low, clear identification of cycling/quiescent clones; subtype-level separation robust. Confounding: Low, within-patient subclonal variation explored with consistent interpretation. Reporting bias: Low, exhaustive figures, raw data and code availability in GEO (GSE118389).
Kim et al. 2023 [18]	Exploratory study using scRNA-seq and scATAC-seq on 2 male ER+ breast cancer biopsies to define transcriptional and enhancer landscapes	Low	OHAT adapted to single-cell multi-omic tumor studies	Selection bias: Low, fresh biopsies immediately processed with high-quality nuclei and paired chromatin profiles. Performance bias: Low, standardized 10x Genomics protocols with quality control at each step. Detection bias: Low, integration of transcriptional and epigenetic signals with consistent enhancer-gene link inference. Confounding: Moderate, only two samples included; however, within-sample robustness and enhancer-level insights mitigate risk. Reporting bias: Low, detailed supplementary data, code available on request.
Gao et al. 2024 [19]	In vitro study using TNBC HCC1143 cell line treated with paclitaxel at 24 h and 72 h, with scRNA-seq, pseudotime, and GRN analyses	Low	SYRCLE (adapted to in vitro perturbation studies with single-cell transcriptomics)	Selection bias: Low, well-controlled cell line model with multiple time points and biological replicates. Performance bias: Low, homogeneous treatment conditions; internal controls and replicability ensured. Detection bias: Low, transcriptomic dynamics analyzed with SCENIC and pseudotime validated by transcription factor expression. Confounding: Low, mechanistic interpretation based on gene regulatory network dynamics. Reporting bias: Low, complete results with figures, raw data available.
Tzeng et al. 2024 [20]	Integrative study using scRNA-seq, spatial transcriptomics, and multi-omics (IHC, proteomics) on 25 fresh breast tumor biopsies and public datasets	Low	ROBINS-I + STROBE-Omics framework	Selection bias: Low, prospective collection of 25 samples, plus external validation from TCGA and GEO. Performance bias: Low, robust multi-modal cross-validation including spatial and IHC data. Detection bias: Low, integration pipelines described in detail; signal reproducibility validated. Confounding: Low, analyses adjusted for tumor subtype, immune infiltration, and spatial clusters. Reporting bias: Low, transparent reporting with open access to data and code.
Yuan et al. 2023 [21]	Multi-modal integrative study using in vitro assays (spheroids), scRNA-seq, proteomics, and in silico analysis across 3132 patient datasets (TCGA, METABRIC, GEO)	Moderate	OHAT + STAIR checklist for multi-omics validation	Selection bias: Moderate, mixed sources (cell lines, public datasets); stemness markers may vary by platform. Performance bias: Low, functional validation via spheroids and proteomic correlation. Detection bias: Moderate, indirect inference of immune–stemness interactions; possible overfitting in gene signature correlation. Confounding: Moderate, immune exclusion and poor prognosis association inferred from correlations, not causality. Reporting bias: Low, complete datasets and results provided; methods clearly outlined.
Zhao et al. 2023 [22]	Multi-omics in silico study integrating CNV, methylation, mutation, transcriptome, and scRNA-seq data from 960 TCGA samples and independent cohorts	Moderate	OHAT + STAIR (Standards for Reporting Omics Research) checklist	Selection bias: Moderate, selection based on TCGA completeness and availability of matched omics layers; independent cohort used for validation. Performance bias: Low, novel REO and DDE methods described and systematically applied. Detection bias: Moderate, lack of spatial resolution; subtypes inferred indirectly from data-driven clusters. Confounding: Moderate, potential patient-level heterogeneity and lack of adjustment for microenvironment variability. Reporting bias: Low, methods and supplemental data well-documented and reproducible.
Zhao et al. 2025 [23]	Integrative spatial multi-omics study using 16 S rRNA sequencing, RNAscope (FISH/CISH), GeoMx DSP, and proteomics on 15 breast tumors	Low	ROBINS-I + STROBE-Omics	Selection bias: Low, spatial mapping performed on fresh tissues with rigorous sectioning (*n* = 60); inclusion of both tumor core and invasive front. Performance bias: Low, multimodal validation (RNA, protein, microbial detection) cross-checked across platforms. Detection bias: Low, consistent spatial colocalization of Fusobacterium nucleatum with transcriptomic shifts. Confounding: Low, associations with MAPK signaling and VEGFD expression controlled for stromal composition. Reporting bias: Low, transparent methodology, raw spatial and proteomic data available in repository.
Regner et al. 2024 [24]	Multi-omics study on 12 primary tumors, 4 normal tissues, and multiple cell lines using scRNA-seq, scATAC-seq, inferCNV, and enhancer-gene modeling	Low	OHAT + STROBE-Omics adapted for single-cell enhancer studies	Selection bias: Low, primary tumors and matched normal samples clearly described; consistent nuclei isolation and sequencing protocols. Performance bias: Low, enhancer-to-gene logic modeled using statistically robust LMMs across samples. Detection bias: Low, multi-modal correlation of expression and chromatin accessibility. Confounding: Low, basal-like subtype explored in depth with transcriptional validation. Reporting bias: Low, high reproducibility, with full data and methods deposited.
Song et al. 2024 [25]	Multi-omics study combining scRNA-seq (*n* = 9 tumors), metabolomics (*n* = 1111 plasmas), and AI-guided prediction for early TNBC detection and response stratification	Low	ROBINS-I + STARD (adapted for diagnostic biomarker research)	Selection bias: Low, well-stratified TNBC cohorts with internal and external validation. Performance bias: Low, metabolomics profiling via both targeted and untargeted approaches; supervised learning tested with cross-validation. Detection bias: Low, inosine/uridine validated as predictive markers; Treg axis confirmed via scRNA signature and A2AR pathway activity. Confounding: Low, diagnostic classifier adjusted for known clinical covariates. Reporting bias: Low, transparent AI pipeline, biomarker data and validation sets made available.
Zhang et al. 2024 [26]	Single-cell study using scRNA-seq (5 TNBC samples), SCENIC, CellChat, and functional assays to define epithelial subtypes and immunomodulatory roles	Low	OHAT + STROBE-Cell	Selection bias: Low, use of freshly resected TNBC tumors with quality control and annotation. Performance bias: Low, integration of gene regulatory networks and intercellular communication robustly validated in vitro. Detection bias: Low, ANXA3 + subtype consistently linked to immune suppression (T-cell inhibition via XBP1 and FOS). Confounding: Low, careful modeling of interactions within epithelial–immune–stromal compartments. Reporting bias: Low, detailed figures, pipeline code, and raw data provided in supplement.
Zhou et al. 2021 [27]	Single-cell transcriptomic and regulatory network analysis on 6 TNBC patients (epithelial + normal cells) using CNV inference, CellPhoneDB, and GRNs	Low	OHAT + STROBE-Cell	Selection bias: Low, primary tumors freshly dissociated; cell type annotation using CNV and lineage markers. Performance bias: Low, rigorous GRN analysis (centrality, regulon activity) across TNBC molecular subtypes. Detection bias: Low, strong subtype-specific signals (e.g., ETV6) cross-validated with immune–tumor interaction data. Confounding: Low, integrated TNBC–macrophage crosstalk analysis supports findings. Reporting bias: Low, all data made publicly available (GEO), pipelines described.
Janesick et al. 2023 [28]	Prospective observational study using human breast tumor specimens (multi-subtype cohort, *n* = 35) analyzed by spatial transcriptomics, scRNA-seq, and RNA-FISH	Low	ROBINS-I	Bias due to confounding: low (subtype stratification and standardized processing). Selection bias: low (well-defined inclusion and pathological confirmation). Performance bias: low (automated sequencing, spatial registration reproducible). Detection bias: low (validated integration pipelines and spatial correlation metrics). Attrition bias: not applicable (cross-sectional design). Reporting bias: low (open data, full transparency of code and raw matrices). Overall risk: low due to methodological transparency, reproducibility, and independent validation.
Wang et al. 2024 [29]	Prospective observational study using human breast tumor samples (*n* = 47) analyzed by Visium ST, mIF and scRNA-seq integration	Low	ROBINS-I	Bias due to confounding: low (standardized sample collection, clinical stratification). Selection bias: low (well-defined inclusion criteria, pathological verification). Performance bias: low (use of automated sequencing and imaging platforms). Detection bias: low (validated bioinformatic pipeline with cross-cohort replication). Attrition bias: not applicable (single-timepoint datasets). Reporting bias: low (transparency of methods, data availability via GEO). Overall risk: low due to robust design and multi-layer validation against external datasets.
Wu et al. 2021 [30]	Cross-sectional single-cell and spatial transcriptomic study on human breast cancer tissues (multi-subtype; *n* ≈ 100 000 cells) integrating scRNA-seq, Visium spatial mapping, and immunohistochemistry	Low	Adapted STROBE + OHAT for omics observational studies	Selection bias: low (multi-center sampling, inclusion of diverse molecular subtypes). Performance bias: low (standardized scRNA-seq and spatial protocols). Detection bias: low (validated computational pipelines and quality control thresholds). Attrition bias: not applicable (cross-sectional design, no follow-up). Reporting bias: low (full datasets publicly available; code and metadata shared). External validity: high (multi-subtype representation, translational relevance). Overall risk judged low due to methodological transparency, robust integration, and reproducibility.

### Data collection process

Data from the included studies were extracted independently by two reviewers using a predefined extraction form in Excel. The data collection process included full-text review and verification of supplementary materials. Disagreements or unclear extractions were resolved by consensus or third-party review. No automation tools were used. When information was missing or ambiguous, we attempted to infer from context or supplementary datasets; otherwise, it was marked as “not reported.”

A qualitative synthesis approach was employed to integrate findings across heterogeneous single-cell and spatial omics studies. Two reviewers (I.B. and O.L.) independently extracted key variables, including study design, sample type, technology platform, analysis pipeline, biological focus, and main outcomes.

A coding framework was developed inductively through iterative reading of the extracted data, grouping findings under three main dimensions: (i) spatial heterogeneity, (ii) temporal heterogeneity, and (iii) tumor–microenvironment interactions. Codes were refined through constant comparison until thematic saturation was achieved.

Double-coding was performed on all studies, and discrepancies were resolved by consensus with a senior reviewer (S.L.). Inter-rater agreement for thematic coding reached 0.89 (Cohen’s κ), indicating high consistency.

Study quality and evidence level (as detailed in Tables 1 and 2) were considered when weighting the relative strength of each theme, ensuring that higher-quality evidence exerted greater interpretive influence during synthesis.

### Data items

The main outcome of interest was the characterization of spatial and/or temporal intratumoral heterogeneity in breast cancer. This included descriptions of subclonal structures, cell lineage trajectories, spatial niches, and tumor–microenvironment interactions. All relevant results relating to heterogeneity domains, regardless of measurement technique or analysis time point, were considered.

We extracted the following variables: author and year, sample origin and type, technology used (scRNA-seq, spatial omics, proteogenomics), study objective, presence of spatial and/or temporal analysis, microenvironment characterization, key findings, level of evidence (based on data depth and validation), and risk of bias. Assumptions were made conservatively when data were incomplete or not explicitly stated.

### Risk of bias assessment

Risk of bias was independently assessed by two reviewers (I.B. and O.L.) using tools tailored to each study design. Specifically, we applied ROBINS-I for observational cohort and diagnostic biomarker studies [31], SYRCLE for in vitro mechanistic studies [32], OHAT (adapted) for in silico or single-cell mechanistic studies [33], and STROBE/STAIR alignment criteria for multi-omics integration studies [34]. Each domain, including selection bias, performance bias, detection bias, confounding, and reporting bias, was evaluated separately. An overall risk of bias rating (low, moderate, or high) was assigned for each study. Discrepancies were resolved through discussion and consensus. In cases where risk assessments required interpretation of supplemental or raw data (e.g., pipelines or in silico outputs), additional reviewer validation was conducted.

### Effect measures

As this review did not aim to evaluate treatment effects quantitatively, no standardized effect measures (e.g., risk ratio or mean difference) were used. Instead, we synthesized results descriptively based on thematic convergence and biological relevance. Where applicable, effect sizes (e.g., AUCs for classifiers) were reported narratively.

### Synthesis methods

Given the marked heterogeneity in study designs, platforms (scRNA-seq vs. diverse spatial technologies), outcomes, and reporting, we did not re-analyze raw data nor conduct a quantitative meta-analysis. Instead, we performed a structured narrative synthesis and a conceptual integration explicitly anchored to the included evidence.

### Reporting bias assessment

We assessed the risk of selective reporting by evaluating the completeness of data presentation (e.g., presence of supplementary tables, raw data access, code availability). Studies without accessible raw data or with vague methodological descriptions were flagged. However, no formal funnel plot or statistical test was applied, given the descriptive nature of the synthesis.

### Certainty assessment

We did not apply GRADE or equivalent certainty frameworks due to the heterogeneity and qualitative nature of the included studies. Nevertheless, we qualitatively assessed certainty based on study size, validation approaches (e.g., in vitro/in vivo), and data reproducibility. High-certainty evidence was defined by consistent findings across independent cohorts and multi-omic validations.

## Results

### Study selection

A total of 1037 records were identified through database searches. After the removal of 94 duplicates, 943 records were screened by title and abstract. Of these, 875 were excluded based on irrelevance to the topic. 68 full-text articles were assessed for eligibility, of which one could not be retrieved. Ultimately, 48 reports were excluded for the following reasons: (i) lack of focus on intratumoral heterogeneity or tumor–microenvironment interactions (*n* = 16); (ii) preclinical studies without human validation (*n* = 13); (iii) bulk-only omics analyses or studies with incomplete methodological description preventing reproducibility (*n* = 10); and (iv) secondary literature such as reviews, editorials, or protocols without results (*n* = 9). This process led to the inclusion of 19 original studies in the final synthesis.

The following results are structured around three complementary dimensions, spatial heterogeneity, temporal dynamics, and tumor–microenvironment interactions, directly reflecting the knowledge gaps identified in the Introduction and addressing the stated objectives of this systematic review.

**Fig. 1:**
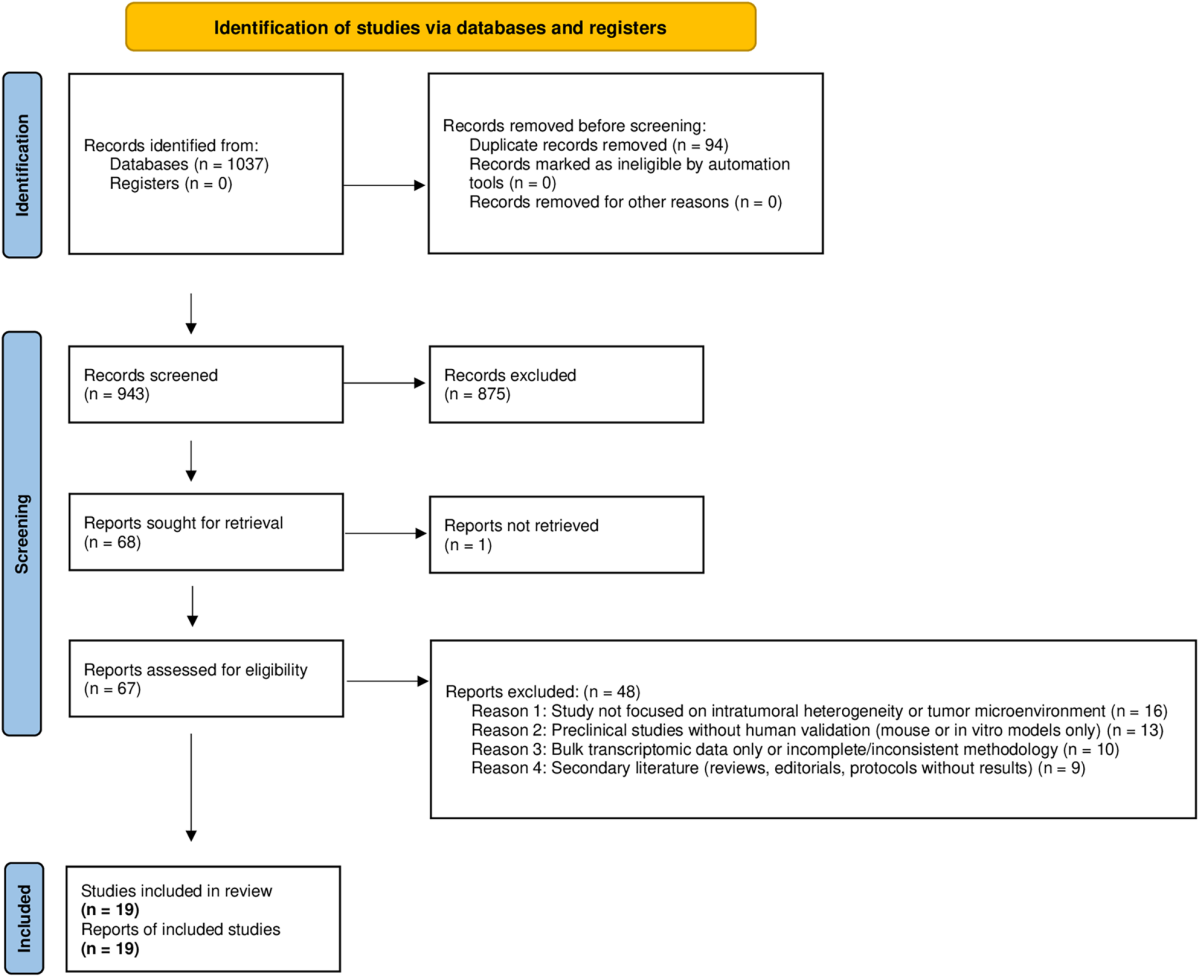
PRISMA 2020 flow diagram summarizing study identification, screening, eligibility assessment, and inclusion steps. *The diagram follows the official PRISMA-2020 format*,* with exclusion reasons grouped into standardized blocks (e.g.*,* irrelevant topic*,* non-human data*,* bulk-level analyses*,* or secondary literature). An editable version of the official PRISMA 2020 form is provided as Supplementary file 1 to ensure transparency and reproducibility*

The full study selection process, including identification, screening, eligibility assessment, and final inclusion, is depicted in Fig. 1.

### Characteristics of included studies

The 19 included studies, published between 2018 and 2025, collectively analyzed human breast tumor samples using single-cell RNA sequencing (scRNA-seq), spatial transcriptomics, or multi-omics integration approaches. Sample sizes ranged from very small exploratory cohorts (*n* = 2 biopsies) to large-scale atlases profiling over 400,000 cells from 181 tumors. While TNBC remained the predominant subtype across the literature, several studies also included ER+, HER2+, or mixed tumor cohorts, improving subtype representativeness. 7 studies incorporated strict spatial-omics platforms (e.g., Visium, GeoMx, multiplex spatial imaging), 9 examined temporal dynamics through pseudotime or treatment-response analyses, and 14 explicitly characterized tumor–microenvironment interactions.

These characteristics, including design, subtype, technologies, and spatial/temporal focus, are summarized comprehensively in Table 1.

### Risk of bias

Risk of bias was assessed using tools adapted to study design: ROBINS-I for cohort studies and diagnostic biomarker research, OHAT for in silico and single-cell studies, SYRCLE for in vitro analyses, and STROBE/STAIR alignments for multi-omics studies. Eleven studies were judged to have low overall risk of bias, while five had moderate risk due to limitations in representativeness, validation, or methodological consistency. Notably, reporting bias was low across all studies, with most providing full datasets, pipelines, and raw data availability.

A detailed synthesis of risk-of-bias ratings across methodological domains for each included study is provided in Table 2.

Given the heterogeneity of study designs, we adapted existing bias assessment frameworks to the omics context. ROBINS-I was used for human observational cohorts, SYRCLE’s tool for preclinical in vitro or in vivo experiments, OHAT for in silico multi-omics analyses, and STROBE/STAIR guidelines for study design quality. Domains such as confounding, selection bias, measurement bias, and reporting bias were retained, while intervention-specific domains (e.g., deviations from intended interventions) were reformulated as “platform or pipeline consistency.” Risk levels (low, moderate, high) were assigned according to transparency of methods, presence of validation, and reproducibility of computational pipelines. We acknowledge that these tools were originally developed for clinical or experimental interventions; thus, their application to omics data introduces conceptual limitations, particularly regarding causal inference and inter-study comparability. Nevertheless, this adapted framework ensured structured and reproducible quality assessment across diverse omics studies.

### Overview of heterogeneity dimensions

#### Spatial Heterogeneity

Thirteen studies explored spatial heterogeneity at various scales. High-resolution spatial transcriptomics and spatial proteogenomics (e.g., Tzeng et al., Zhao et al.) [20, 23] revealed the existence of tumor subclones localized within distinct niches, often interacting with immune or stromal components. Fusobacterium nucleatum was spatially colocalized with oncogenic signaling in Zhao et al. [23], while several studies (e.g., Zhang et al., Karaayvaz et al.) [17, 26] mapped epithelial subtypes with spatial restriction and functional divergence.

Recent high-resolution spatial transcriptomic studies [28–30] further delineated conserved epithelial–stromal architectures across breast cancer subtypes, revealing spatial coupling of EMT-like epithelial cells with CAF-rich and macrophage-dense niches driving immune exclusion and metabolic adaptation.

These findings suggest that spatial omics could inform biopsy targeting strategies, ensuring that resistant or immune-excluded niches are adequately sampled for diagnostic and therapeutic decision-making.

#### Temporal dynamics

Nine studies investigated temporal dynamics, either through treatment timelines (e.g., Gao et al.) [19], pseudotime inference (e.g., Cheng et al., Davies et al.) [13, 14], or comparisons of treatment-naïve and treated tumors. Dynamic remodeling of the transcriptomic landscape, emergence of resistant clones, and shifting cell–cell communication profiles were consistently reported, underscoring the fluidity of clonal evolution.

Clinically, temporal monitoring through longitudinal sampling may support adaptive therapy approaches, where treatment intensity or sequencing is adjusted in real time to counteract emerging resistant clones.

#### Microenvironmental Interactions

All but one study [24] characterized the tumor immune microenvironment. Consistent findings included the role of M2-like macrophages in immunosuppression (e.g., Jiang et al., Cheng et al.) [13, 16], the spatial segregation of immune–tumor interfaces, and the identification of immune-excluded subclones. Several studies, including Song et al. and Yuan et al. [21, 25], linked metabolic or stemness features to immunomodulatory pathways via scRNA-seq and AI-driven integration.

These insights highlight the potential of spatially resolved immune biomarkers, such as macrophage or Treg niches, to stratify patients for immunotherapy and predict checkpoint inhibitor efficacy.

### Methodological trends and innovations

Several analytical pipelines emerged across studies. Most scRNA-seq datasets employed Seurat for clustering and CNV inference; spatial analyses relied on 10x Visium, GeoMx DSP, or RNAscope validation. Multi-modal studies frequently integrated enhancer–gene mapping (e.g., Regner et al.) [24] or metabolic layers (e.g., Song et al.) [25]. Across the review, there was a notable shift toward functional validation (in vitro or in vivo), especially in post-2022 studies.

### Evidence synthesis

Despite the methodological heterogeneity across studies, the synthesis revealed several converging themes. Subclonal plasticity emerged as a pervasive feature, frequently associated with epithelial–mesenchymal transition traits, cycling cellular states, and transcriptional programs indicative of immune evasion. The spatial organization of these subclones often mirrored the structure of the tumor microenvironment, particularly through localization within vascular niches and immune-excluded regions. Temporal analyses further uncovered evolutionary trajectories characterized by lineage bifurcation and therapy-induced selection pressures, notably in response to chemotherapy or immune checkpoint blockade. Moreover, patterns of inter-clonal communication and interactions between tumor and immune cells consistently correlated with increased disease aggressiveness and therapeutic resistance.

These observations support a model of breast cancer as a highly dynamic and structured ecosystem, where space and time jointly shape phenotypic plasticity and clinical behavior.

## Discussion

The results of this review directly address the knowledge gaps outlined in the Introduction. By systematically mapping spatial organization, temporal evolution, and microenvironmental crosstalk, we provide an integrated resolution of the three major uncertainties previously identified: the dynamic nature of clonal evolution, the role of spatial niches in resistance, and the methodological variability across single-cell and spatial studies.

This systematic review synthesized 19 original studies published between 2018 and 2025 investigating spatial and temporal intratumoral heterogeneity in human breast cancer using single-cell and spatial omics technologies. Despite methodological variability across platforms, study designs, and analytical pipelines, the collective body of evidence revealed strikingly convergent biological patterns and reinforced the importance of high-resolution profiling in delineating tumor evolution and ecosystem complexity.

Unlike previous reviews that have summarized single-cell or spatial studies in isolation [9, 11], the present work provides the first systematic and evidence-weighted synthesis explicitly integrating spatial and temporal dimensions of intratumoral heterogeneity in breast cancer. This dual-axis framework enables the identification of conserved ecological programs, linking spatial immune exclusion with temporal clonal adaptation, that were not jointly captured in prior literature.

Moreover, while most included studies converged on common themes such as epithelial–mesenchymal plasticity and immune evasion, this review also highlights where evidence remains incomplete or conflicting. For instance, discrepancies persist in defining ‘cellular states’ across clustering algorithms and in the limited longitudinal validation of inferred trajectories.

### General interpretation of findings in light of other evidence

The reviewed studies consistently demonstrated that breast tumors are composed of transcriptionally and functionally distinct subclones whose behaviors are shaped by their spatial location and temporal context. Subclonal plasticity was a hallmark feature across nearly all datasets, with several studies highlighting a continuum of epithelial-to-mesenchymal transition (EMT), quiescence-cycling dynamics, and immune evasion as key signatures of aggressive or therapy-resistant states (e.g., Karaayvaz et al., Zhang et al., Gao et al.) [15, 17, 26].

Spatial architectures actively modulate signaling and therapy response, underscoring their translational relevance for precision diagnostics. Clinically, such spatial compartmentalization could inform biopsy targeting, ensuring that resistant or immune-excluded niches are sampled rather than missed by random core biopsies. This highlights the translational potential of spatial omics in guiding precision diagnostics.

Integrative spatial studies have now demonstrated that such architectures are not random but conserved across subtypes, with recurrent spatial programs linking epithelial plasticity to localized immunosuppression [28–30]. These findings reinforce the concept of tumor ecosystems organized into spatially restricted ecological niches that influence both evolution and therapy resistance.

Temporally, studies using pseudotime, perturbation experiments, or comparisons between naïve and treated tumors revealed dynamic lineage bifurcations, transcriptional reprogramming under therapy, and selection of resistant clones (e.g., Cheng et al., Gao et al., Davies et al.) [13, 14, 19]. Several works also demonstrated the temporal activation of interferon signaling, metabolic rewiring, or enhancer reconfiguration following drug exposure.

**Fig. 2:**
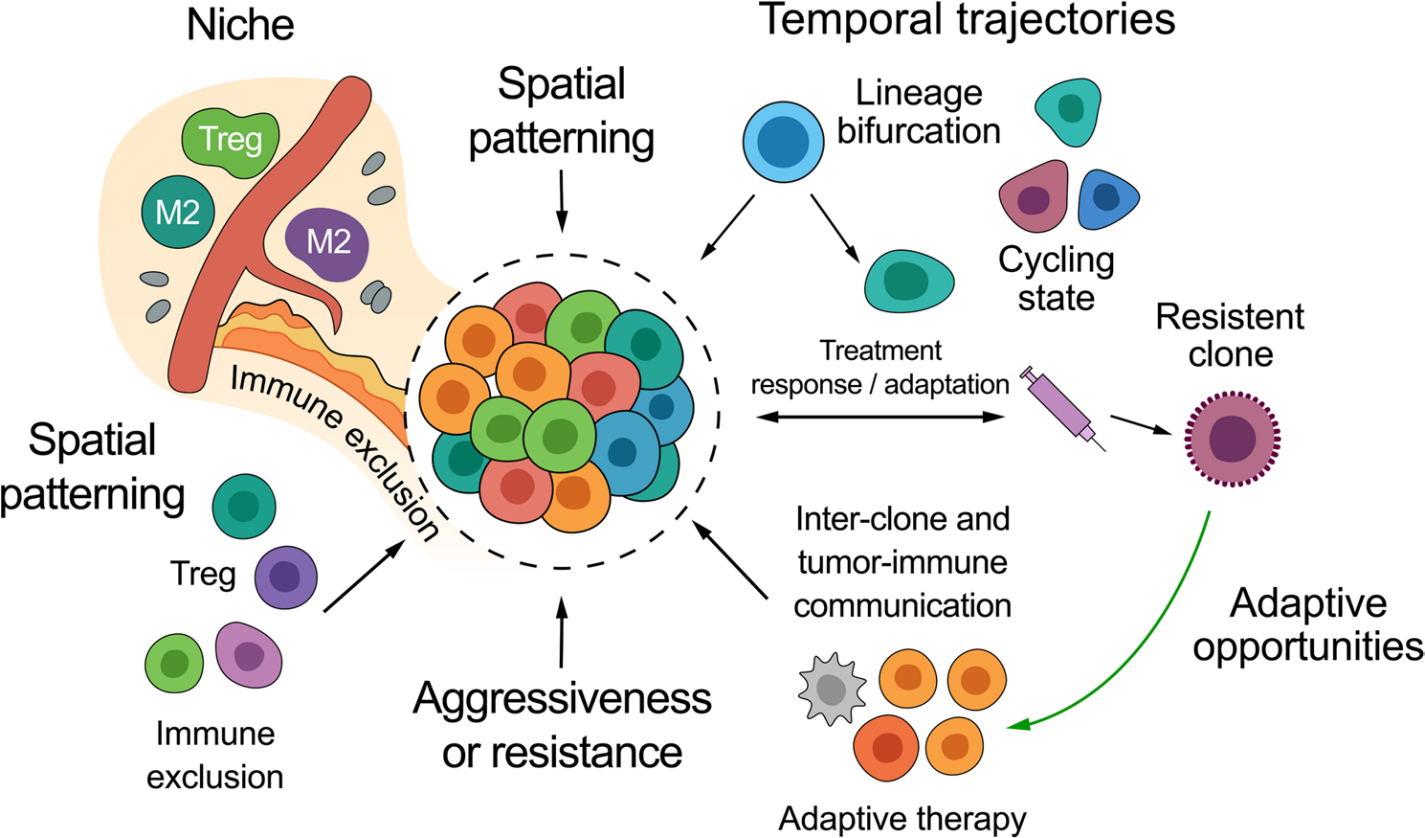
Conceptual model of spatial and temporal intratumoral heterogeneity in breast cancer. *The diagram illustrates how spatial patterning (immune exclusion*,* stromal barriers*,* regulatory T cells*,* and M2 macrophages) and temporal trajectories (lineage bifurcation*,* cycling states*,* treatment-induced resistant clones) converge to drive tumor aggressiveness and therapeutic resistance. In parallel*,* interclonal and tumor–immune communication pathways are highlighted as key mediators of immune suppression. Importantly*,* the model also emphasizes adaptive opportunities: spatiotemporal analyses may guide biopsy targeting*,* patient stratification*,* and adaptive therapy strategies aimed at overcoming resistance. This figure is an evidence-based conceptual synthesis built from the studies included in this review; it does not present new data. Additional measurable spatial features such as cell–cell proximity probabilities*,* spatial entropy indices*,* or immune niche density can be quantified using emerging computational frameworks (e.g.*,* SpatialQPFs or entropy-based measures) to operationalize the conceptual processes illustrated in this figure*

Importantly, inter-clonal and tumor–immune communication, mapped via ligand–receptor analyses, gene regulatory networks, or spatial proximity, was consistently associated with poor prognosis, immune suppression, or immunotherapy resistance. These findings build upon and expand prior bulk-level observations by revealing the precise cellular mediators and niches underlying immunomodulation. Each conceptual component illustrated in Fig. 2 (immune exclusion, lineage bifurcation, therapy-induced resistant clones, and interclonal communication) is directly supported by the studies cited in this Sects. [13–15, 26, 28–30]. To synthesize these dimensions, we propose a conceptual model (Fig. 2) illustrating how spatial patterning, temporal trajectories, and interclonal/tumor–immune communication converge to shape tumor aggressiveness, resistance, and therapeutic opportunities. Our conceptual diagram therefore summarizes, rather than re-derives, relationships observed across heterogeneous studies, aiming to provide a clinically interpretable scaffold for spatial and temporal heterogeneity while also serving as a conceptual bridge between mechanistic studies and clinical translation.

Spatial integration analyses confirmed that ligand–receptor interactions between tumor cells, CAFs, and macrophages are spatially patterned, forming micro-domains of immune exclusion consistent with the ecological heterogeneity model supported by Janesick et al. and Wang et al. [28, 29].

Beyond biological insights, recent methodological advances have introduced quantitative frameworks to formally characterize spatial relationships and ecological complexity in tumors, including SpatialQPFs for modeling cell–cell spatial interaction fields and entropy-based metrics for quantifying spatial disorder. Although these approaches remain underused in breast cancer single-cell and spatial omics research, they offer promising tools that could complement high-resolution molecular profiling by providing objective, reproducible spatial descriptors [35].

A notable limitation across current single-cell and spatial studies is the predominance of qualitative or semi-quantitative descriptions of spatial organization (e.g., “immune exclusion,” “ecological niches”) rather than standardized quantitative spatial metrics. Emerging frameworks such as SpatialQPFs and entropy-based measures can formally quantify spatial autocorrelation, spatial clustering, or microenvironmental complexity. Their limited adoption restricts cross-study comparability and highlights the need for unified quantitative spatial descriptors in future spatial omics research [36].

Taken together, the convergence of epithelial–mesenchymal plasticity, metabolic rewiring, and immune evasion observed across single-cell and spatial studies suggests that breast cancer progression is governed by a limited set of evolutionary programs repeatedly selected under therapeutic and microenvironmental pressures. These recurrent axes, clonal diversification, microenvironmental adaptation, and temporal reprogramming, constitute a unifying biological framework linking intratumoral heterogeneity to clinical behavior. Recognizing these conserved mechanisms could enable the design of therapeutic strategies targeting evolutionary dependencies rather than isolated molecular alterations.

Despite the depth of mechanistic insight provided by single-cell and spatial omics, a substantial translation gap remains between high-resolution profiling and real-world therapeutic decision-making. Most spatial or lineage-resolved signatures identified across studies lack prospective clinical validation, and their integration into routine practice is limited by cost, scalability, and the absence of standardized analytical frameworks. Moreover, while subclonal plasticity, immune-excluded niches, and therapy-induced trajectories are now well-characterized at the mechanistic level, it is not yet clear how these features should directly inform drug selection, sequence optimization, or patient stratification outside research settings. Bridging this gap will require harmonized spatial metrics, clinically annotated longitudinal cohorts, and prospective trials incorporating single-cell or spatial readouts to determine which mechanistic features are truly actionable.

It should be noted that most of the studies included in this review focused on TNBC, reflecting the research bias of the field rather than a selection bias of this work. TNBC has historically served as a model system for studying plasticity, immune exclusion, and therapy resistance due to its pronounced heterogeneity and lack of targeted treatments. Consequently, while some mechanisms described here may be more thoroughly characterized in TNBC, similar biological principles are increasingly observed across ER + and HER2 + subtypes as single-cell and spatial datasets expand [30].

With the inclusion of three additional high-resolution spatial-omics studies [28–30], this review now encompasses nineteen studies, thereby improving the molecular and clinical representativeness of the dataset. These recent contributions extend the evidence base beyond TNBC to include luminal and HER2-positive tumors, allowing cross-subtype comparisons of spatial and temporal programs. Nevertheless, TNBC remains predominant among published single-cell datasets, reflecting a research rather than selection bias. This imbalance should be considered when interpreting generalizability, although the convergence of spatial–temporal mechanisms across molecular contexts supports the broader applicability of the synthesized framework.

### Limitations of the evidence base

While the reviewed studies provide deep mechanistic insight, certain limitations warrant consideration. First, despite high-resolution technologies, many datasets suffer from limited sample sizes, especially in rare subtypes or male breast cancer. The generalizability of single-cell atlases derived from a handful of patients remains uncertain. Second, validation methods varied substantially across studies; some offered only in silico inference without experimental support, while others included in vitro or murine models with limited translational fidelity. Third, not all platforms offered spatial or temporal resolution, and in some cases, annotations of subclonal identity were inferred rather than experimentally confirmed. Moreover, technical biases such as cell dropout, dissociation artifacts, and batch effects remain nontrivial in single-cell omics and may contribute to noise or overfitting.

In addition, potential publication bias should be acknowledged, as single-cell studies with clearer or positive findings are more likely to be published. This bias, together with the persistent under-representation of ER+ tumors compared with TNBC in current datasets, may limit the comprehensive generalizability of the synthesized evidence.

Additionally, many studies lacked longitudinal clinical follow-up, which hinders the direct correlation between inferred cellular trajectories and patient outcomes over time. Furthermore, key concepts such as “subclonal plasticity” or “cellular states” were variably defined across studies, limiting harmonization and meta-level synthesis. Establishing standardized ontologies and shared reporting frameworks will be critical for enhancing reproducibility and interpretability in future single-cell and spatial omics research.

Although spatial transcriptomics has expanded since 2021, only a few studies [28–30] have combined spatial, temporal, and single-cell resolution simultaneously, underscoring the need for multi-modal longitudinal designs.

### Limitations of the review process

Although this review followed a rigorous and pre-registered PRISMA-based protocol with transparent inclusion and exclusion criteria, several limitations must be acknowledged. No formal meta-analysis was performed due to the heterogeneity of data types and outcomes. The synthesis was therefore narrative and relied on structured tabulation and visual inspection. Furthermore, some relevant articles might have been missed if not indexed with standard terms or if they lacked open-access availability. Lastly, our risk-of-bias assessments, while comprehensive, were limited by the granularity of reporting in original studies and by adaptations of tools (e.g., OHAT, STROBE-Omics) to emerging single-cell methodologies.

### Implications for practice, policy, and research

The findings of this review carry several implications. Clinically, single-cell and spatial profiling are poised to redefine biomarker discovery, enabling detection of resistant subpopulations or immune escape niches that would be missed in bulk sequencing. This may inform personalized therapeutic strategies, particularly in aggressive or immunologically “cold” tumors. The identification of therapy-induced transcriptional trajectories and enhancer rewiring also raises the possibility of rational drug sequencing to pre-empt clonal adaptation [13, 37–39].

From a research perspective, our synthesis supports the continued integration of spatial and temporal resolution in cancer studies. Longitudinal sampling, lineage tracing, and AI-guided modeling of evolution are promising avenues for future exploration. In addition, spatially-informed adaptive therapy strategies could be envisioned, where treatment intensity or sequencing is adjusted in real time based on the emergence of resistant subclones detected by high-resolution profiling. Moreover, there is a need for unified analytical frameworks and better reporting standards, particularly for integrating spatial data with transcriptomics and for defining cell states in functional terms.

Finally, future work should prioritize cross-study harmonization, experimental validation in clinically annotated cohorts, and the translation of single-cell insights into scalable diagnostics or therapeutic interventions. With the increasing accessibility of multi-modal platforms and computational pipelines, the field is well-positioned to move from description to intervention, targeting not only tumor cells but the evolving ecologies in which they reside.

## Conclusion

This systematic review underscores the transformative potential of single-cell and spatial omics technologies in elucidating the complexity of intratumoral heterogeneity in breast cancer. Across 19 original studies published between 2018 and 2025, we observed consistent evidence that spatial architecture, temporal dynamics, and subclonal plasticity jointly define tumor behavior and therapeutic response. Subclonal ecosystems shaped by epithelial–mesenchymal transitions, immune evasion, and lineage bifurcation were frequently linked to aggressive phenotypes or resistance mechanisms. Spatially restricted immune niches, vascular territories, and fibroblast-rich zones emerged as key regulators of cellular fate and treatment outcomes. Moreover, the temporal reprogramming of gene expression under therapeutic pressure, particularly involving interferon signaling, metabolic adaptation, and enhancer remodeling, revealed dynamic layers of tumor evolution that are invisible to bulk profiling.

From a clinical and research perspective, our findings call for a paradigm shift in how tumor biology is interrogated and translated. High-resolution profiling should no longer be viewed as exploratory but as essential for identifying actionable targets, tracking resistance trajectories, and tailoring patient-specific therapies. However, the integration of spatial and temporal omics into clinical workflows requires standardization, scalable technologies, and robust validation pipelines. Future studies should aim to harmonize analytical frameworks, leverage longitudinal sampling, and explore real-time single-cell diagnostics. Ultimately, this synthesis reinforces that tumor progression is not merely a genetic phenomenon but a dynamic ecological process, driven by evolving interactions among clones, immune cells, and stromal components, requiring equally adaptive and multidimensional therapeutic strategies.

Although the majority of available evidence currently derives from TNBC datasets, the emerging frameworks of spatial and temporal heterogeneity are broadly applicable across molecular subtypes. Future single-cell and spatial studies in ER + and HER2 + breast cancers will be essential to confirm the generalizability of these mechanisms.

## Supplementary Information

Supplementary Material 1: Supplementary file 1. Supplementary table 1. Glossary of key terms used throughout the manuscript.

## Data Availability

All data analyzed in this systematic review were extracted from previously published and publicly accessible original research articles. The full dataset, including the PRISMA flowchart and extraction tables, is provided directly within the submitted manuscript.
