# Supplementary material for: Spatial and temporal intratumoral heterogeneity in breast cancer: a systematic and conceptual review of single-cell and spatial omics studies
Source: BMC Cancer. 2026 Apr 6;26:639. doi: 10.1186/s12885-026-15928-0 (PMC13188440; doi:10.1186/s12885-026-15928-0)
Supplement: Supplementary file 1 — Supplementary Material 1: Supplementary file 1. Supplementary table 1. Glossary of key terms used throughout the manuscript. [file 12885_2026_15928_MOESM1_ESM.docx]

**Supplementary file 1**

**Identification of studies via databases and registers**

Records removed *before screening*:

Duplicate records removed (n = )

Records marked as ineligible by automation tools (n = )

Records removed for other reasons (n = )

Records identified from*:

Databases (n = )

Registers (n = )

**Identification**

Records screened

(n = )

Records excluded**

(n = )

Reports sought for retrieval

(n = )

Reports not retrieved

(n = )

**Screening**

Reports assessed for eligibility

(n = )

Reports excluded:

Reason 1 (n = )

Reason 2 (n = )

Reason 3 (n = )

etc.

Studies included in review

(n = )

Reports of included studies

(n = )

**Included**

*Consider, if feasible to do so, reporting the number of records identified from each database or register searched (rather than the total number across all databases/registers).

**If automation tools were used, indicate how many records were excluded by a human and how many were excluded by automation tools.

**Supplementary table 1:** Glossary of key terms used throughout the manuscript.

| Term | Definition |
| --- | --- |
| Tumor microenvironment (TME) | The ecosystem of immune, stromal, and vascular cells surrounding tumor cells, influencing growth and therapy response. |
| Immune exclusion | Spatial segregation of immune cells at the tumor periphery preventing effective intratumoral infiltration. |
| Subclonal plasticity | The ability of tumor subclones to switch phenotypic or transcriptional states under selective pressures. |
| Lineage bifurcation | Divergence of tumor cell lineages into distinct evolutionary or functional trajectories. |
